# Harnessing ferroptosis for enhanced sarcoma treatment: mechanisms, progress and prospects

**DOI:** 10.1186/s40164-024-00498-3

**Published:** 2024-03-12

**Authors:** Jing Zeng, Xianghong Zhang, Zhengjun Lin, Yu Zhang, Jing Yang, Pengcheng Dou, Tang Liu

**Affiliations:** 1 Department of Orthopedics, The Second Xiangya Hospital of Central South University, Changsha, 410011 Hunan China; 2 Department of Orthopedics, The Fifth Affiliated Hospital of Xinjiang Medical University, Urumqi, 830000 Xinjiang China

**Keywords:** Ferroptosis, Sarcoma, Mechanism, Prognosis prediction, Drug resistance

## Abstract

Sarcoma is a malignant tumor that originates from mesenchymal tissue. The common treatment for sarcoma is surgery supplemented with radiotherapy and chemotherapy. However, patients have a 5-year survival rate of only approximately 60%, and sarcoma cells are highly resistant to chemotherapy. Ferroptosis is an iron-dependent nonapoptotic type of regulated programmed cell death that is closely related to the pathophysiological processes underlying tumorigenesis, neurological diseases and other conditions. Moreover, ferroptosis is mediated via multiple regulatory pathways that may be targets for disease therapy. Recent studies have shown that the induction of ferroptosis is an effective way to kill sarcoma cells and reduce their resistance to chemotherapeutic drugs. Moreover, ferroptosis-related genes are related to the immune system, and their expression can be used to predict sarcoma prognosis. In this review, we describe the molecular mechanism underlying ferroptosis in detail, systematically summarize recent research progress with respect to ferroptosis application as a sarcoma treatment in various contexts, and point out gaps in the theoretical research on ferroptosis, challenges to its clinical application, potential resolutions of these challenges to promote ferroptosis as an efficient, reliable and novel method of clinical sarcoma treatment.

## Introduction

Sarcoma is a type of malignant tumor originating from mesenchymal tissue and is broadly categorized into two primary groups: bone sarcomas and soft tissue sarcomas. Osteosarcoma, a bone sarcoma, is the most prevalent sarcoma among children and adolescents, with a 5-year survival rate ranging from 60 to 70%. Remarkably, this rate has seen little improvement over the past three decades [1, 2]. On the other hand, soft tissue sarcomas are complex malignancies that include at least 100 different histological and molecular subtypes [3]. The overall 5-year survival rate for soft tissue sarcomas stands at approximately 50% [4]. While local surgical resection coupled with chemotherapy and radiotherapy has demonstrated effectiveness in treating both types of sarcoma, its overall efficacy remains limited, and advancements in novel treatments have been sluggish [5].

**Fig. 1:**
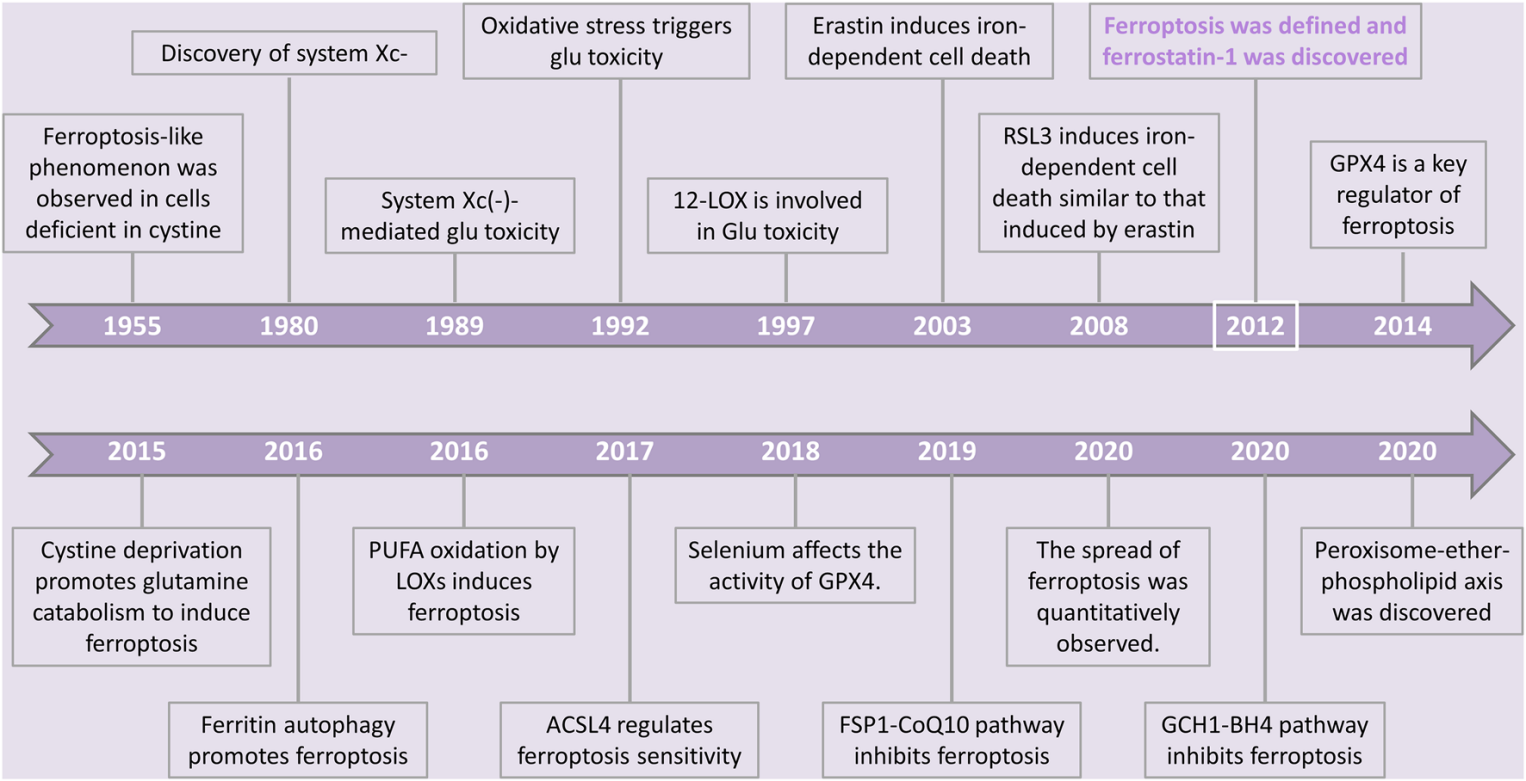
Landmark events related to the development of ferroptosis

Ferroptosis represents a distinctive type of cell demise induced by erastin, which is a kind of oncogenic RAS-selective lethal small molecule (RSL). It is hallmarked by the intracellular accumulation of free iron and lipid peroxides and stands apart from apoptosis, necrosis, and autophagy in terms of morphology, biochemistry, and genetics [6]. Although the term "ferroptosis" was not formally introduced until 2012, with the discovery of the small-molecule inhibitor ferrostatin-1 by Dixon et al. [6], the characteristics of this mode of cell death had been described in earlier research and have continued to evolve since the coining of the term [7]. (Fig. 1). In normal conditions, cells manage stress associated with ferroptosis through the regulation of various antioxidant systems, which can, in turn, serve as targets for interventions aimed at inducing ferroptosis in cells.

Ferroptosis has the potential to be a new and highly effective treatment for sarcoma. Recent research has unveiled a profound connection between ferroptosis and the pathophysiological mechanisms underlying a diverse spectrum of diseases, including cancer, neurological disorders, ischemia–reperfusion injury, kidney damage, and hematological disorders [8]. Notably, ferroptosis is emerging as a novel drug target, offering avenues to overcome chemotherapy resistance and predict disease prognosis in the context of sarcoma treatment [9–11]. Therefore, it is of great importance to study the mechanism underlying ferroptosis and its potential as a sarcoma treatment.

This comprehensive review meticulously delineates the molecular mechanisms that underlie ferroptosis. It distills and deliberates upon recent breakthroughs and advancements in ferroptosis research as a prospective strategy for the prevention and treatment of sarcoma. Furthermore, the review offers a visionary perspective on the clinical applications of ferroptosis, while candidly addressing the limitations and challenges inherent in these findings, providing a roadmap for future research directions.

In summary, ferroptosis stands at the forefront of innovative approaches to sarcoma therapy. Understanding the intricacies of this distinctive form of cell death not only holds the promise of more effective treatments but also the potential to prognosticate disease outcomes, thereby benefiting sarcoma patients and advancing the broader landscape of cancer research.

## Molecular mechanisms underlying ferroptosis

**Fig. 2:**
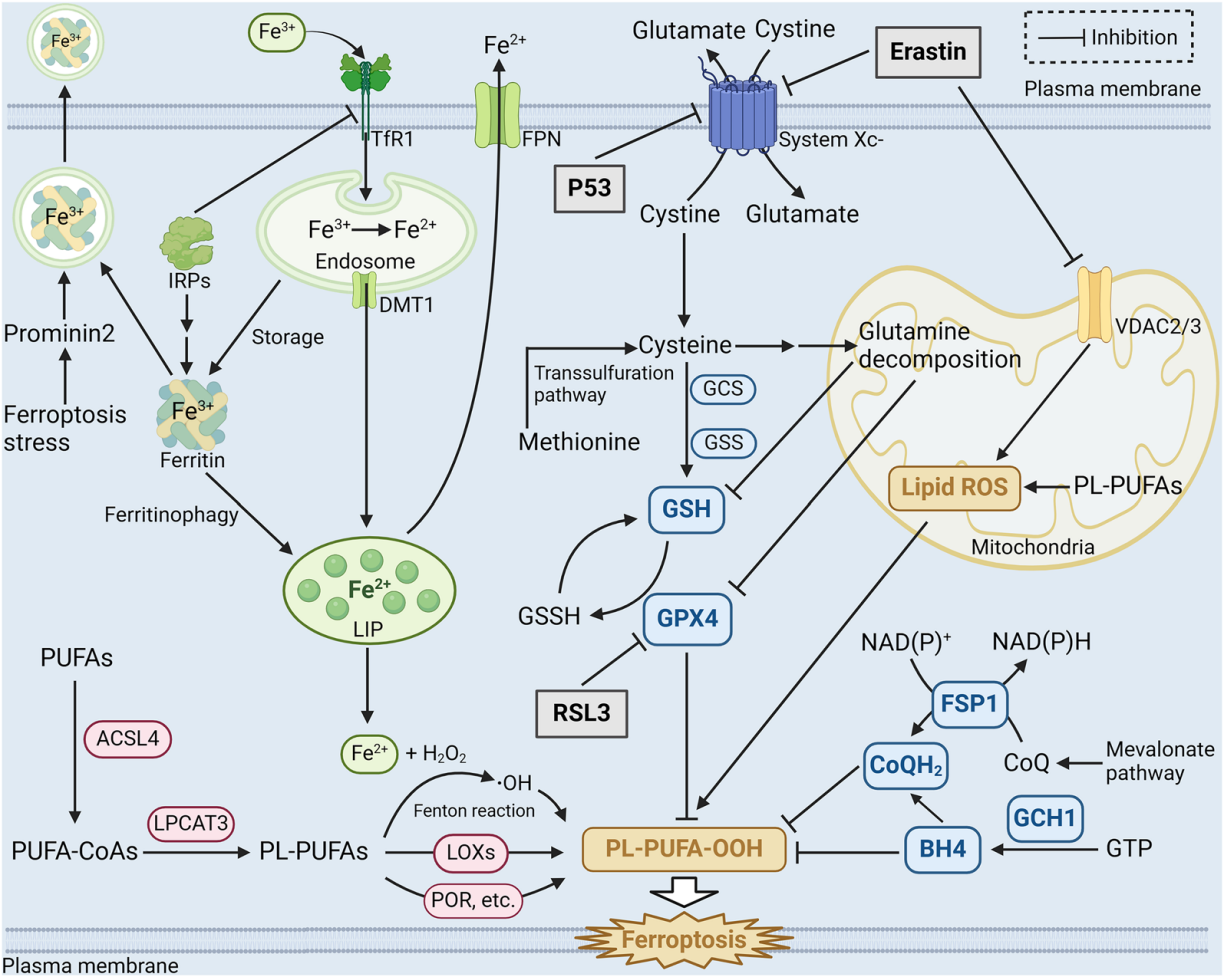
Molecular mechanism of the occurrence and regulation of ferroptosis. PUFAs are modified into PL-PUFAs, which produce lipid peroxides through the Fenton reaction involving Fe^2+^ or catalyzed by iron-dependent lipoxygenases (red), then attack cells to cause ferroptosis. The intracellular iron metabolism (green), three major regulatory pathways (system Xc(−)-GSH-GPX4 pathway, NADPH-FSP1-CoQ10 pathway and GCH1-BH4 pathway. blue) and mitochondrial involvement (orange) together regulate the process of ferroptosis. Three common ferroptosis inducers: erastin, RSL3, and p53 are exemplified (gray). (Created with BioRender.com.)

Ferroptosis, a relatively recent discovery in the realm of cell biology, represents a unique form of regulated cell death. This process hinges on a distinct chemical cascade ignited by the iron-dependent buildup of lipid peroxides. Ferroptosis, in essence, is orchestrated by an intricate interplay of various redox-active enzymes, each with roles in the generation or elimination of free radicals and lipid oxidation products. These molecular actors collectively orchestrate an intracellular redox imbalance, culminating in the cell’s demise. This tightly regulated cell death mechanism operates at multiple hierarchical levels, spanning epigenetic, transcriptional, posttranscriptional, and posttranslational tiers of control. In this orchestrated dance of molecular players, ferroptosis unveils itself as a multifaceted and carefully governed phenomenon in the intricate tapestry of cell biology [12]. (Fig. 2).

### Abnormal iron metabolism initiates ferroptosis

Iron is an essential trace element for cell growth and metabolism and is a component of the catalytic site of many important redox enzymes. However, too much iron can be extremely hazardous to cells and cause oxidative DNA damage [13]. Intracellular iron is stored in a metabolically active pool called the “labile iron pool” (LIP). The LIP can store, export, or consume iron, and most of the iron (> 80%) in cells is available in ferrous form [14, 15].

When dysregulation of intracellular iron leads to abnormal accumulation of free iron, excessive amounts of lipid peroxide are produced, triggering ferroptosis. A study showed that iron chelation therapy attenuated ferroptosis in a rodent model of cerebral ischemia‒reperfusion injury [16]. Intracellular free iron is involved in two main pathways of ferroptosis, the nonenzymatic pathway and the enzymatic pathway. In the nonenzymatic pathway, excess iron generates hydroxyl radicals through the Fenton reaction, and these products enriches the intracellular reactive oxygen species (ROS) pool and promotes the oxidation of polyunsaturated fatty acids (PUFAs, such as arachidonic acid and linoleic acid) to generate lipid peroxides and hydroperoxides, which then attack adjacent PUFAs and trigger chain reactions [17, 18]. When the pathways that inhibit lipid peroxidation in a cell fail, the chain reaction eventually reaches cell membrane lipids, causing structural and functional damage and leading to ferroptosis [19].

Furthermore, iron-dependent lipoxygenase (LOX) serves as a catalyst in the enzymatic pathway, facilitating the production of lipid peroxides, thereby increasing the sensitivity of cells to ferroptosis. Notably, inhibition or knockdown of LOXs can inhibit ferroptosis in specific cell types, which further proves their importance to ferroptosis [20]. Remarkably, a specific lipoxygenase subtype, 12/15-LOX, has been identified as a key player in the oxidation of PUFAs linked to ferroptosis [21]. This observation hints at a potential pivotal role for 12/15-LOX in the regulation of ferroptosis, although further research is needed to solidify this connection [22]. In the context of iron overload, substances like hemoglobin and ferrous ammonium sulfate can also trigger ferroptosis. The mechanisms responsible for their effects involve the activation of distinct LOX protein subtypes, underscoring the diverse roles these enzymes play in driving ferroptotic cell death [23]. Additionally, other iron-dependent enzymes, notably cytochrome P450 oxidoreductase (POR), have been recognized as instigators of lipid peroxidation and ferroptosis under specific conditions [24], further illustrating the intricate network of molecular interactions governing this unique form of programmed cell death.

Hence, the development of ferroptosis can be influenced by interventions at various stages of cellular iron metabolism. Transferrin (TF) and transferrin receptor (TFRC) play crucial roles in this process. TF, binding to nearly all forms of circulating iron under physiological conditions, facilitates the entry of iron ions into cells by recognizing and binding to TFRC. In the SKBR3 and MDA-MB-231 cancer cell lines, ferroptosis induced by compounds like lapatinib and siramesine was observed to be mitigated when TF was knocked down [25]. Similarly, the deletion of TFRC prevented ferroptosis induced by erastin or cystine deficiency [26, 27]. These findings underscore the regulatory influence of TF and TFRC on iron uptake, subsequently affecting the sensitivity of cells to ferroptosis.

The mechanism governing the storage of iron within cells holds significant importance in the context of ferroptosis. Ferritin plays a key role in this process by storing intracellular iron in an inert form, primarily as Fe^3+^. Decreasing the LIP and elevating ferritin levels can be instrumental in preventing ferroptosis [28]. Moreover, in the cytoplasm, the overexpression of ferritin within mitochondria has been observed to thwart ferroptosis induced by compounds like erastin in neural cells [29]. Conversely, ferritin-targeted autophagy, also known as ferritin autophagy (chapter " The ferroptosis propagation”), has the opposite effect, increasing cell susceptibility to ferroptosis [30, 31]. Furthermore, research has revealed that prominin 2 mediates the release of ferritin and iron from cells via exosomes as a protective mechanism against ferroptosis [32]. This suggests that intracellular pathways governing iron degradation and secretion collectively regulate the LIP level, exerting a substantial influence on a cell’s susceptibility to ferroptosis.

Solute carrier family 40 member 1 (SLC40A1), also known as ferroportin (FPN), stands as the sole recognized iron transporter protein residing on mammalian cell membranes, primarily responsible for facilitating iron efflux. The modulation of SLC40A1 plays a pivotal role in the regulation of ferroptosis. Notably, when SLC40A1 is knocked down, it heightens the ferroptotic process. Conversely, the overexpression of SLC40A1 has been observed to decelerate the rate of ferroptosis [25, 33]. Thus, by regulating the outflow of cellular iron, SLC40A1 plays a crucial regulatory role in ferroptosis.

In conclusion, intracellular iron is involved in the formation of lipid peroxides and free radicals through the Fenton reaction or functions at the active sites of enzymes. Through these two functions, intracellular iron is related to ferroptosis. Moreover, modulation of multiple targets in the iron metabolism pathway can promote or prevent ferroptosis by affecting the amount and availability of intracellular iron, and many of the targets affected by iron metabolism have been demonstrated via experiments [34]. These discoveries open up numerous possibilities for leveraging ferroptosis as a therapeutic approach in the treatment of various diseases.

### Lipid peroxidation provides materials essential for ferroptosis

Ferroptosis is characterized by the accumulation of intracellular lipid peroxides and their damaging effects on the cell membrane. Lipids, as the fundamental building blocks of cellular membranes, play a vital role in maintaining normal physiological processes. However, the excessive buildup of lipid peroxides can result in a range of structural and functional impairments within cells, a phenomenon frequently observed in the cells of diseased tissues [19].

When subjected to enzymatic processes or attacked by free radicals, PUFAs give rise to the production of lipid peroxides. For example, during a ROS assault, arachidonic and linoleic acids generate lipid peroxidation products, which trigger ferroptosis [35, 36]. Conversely, it was found that whether the fatty acid β-oxidation (FAO) in mitochondria consumes fatty acids, the formation of lipid droplets isolates PUFAs and protects them [34], or the competitive inhibition of PUFAs by monounsaturated fatty acids [37–39], they can all effectively prevent cells from ferroptosis by reducing the peroxidation of PUFAs. Moreover, PUFAs require modification before they can actively contribute to peroxidation reactions. Lysophosphatidylcholine acyltransferase-3 (LPCAT3) and acyl-CoA synthetase long-chain family member 4 (ACSL4) are recognized as significant regulators of ferroptosis [40–42]. They facilitate the conversion of PUFAs into phospholipid-bound polyunsaturated fatty acids (PL-PUFAs), which can directly participate in lipid peroxidation. Research indicates that phosphatidylcholine and phosphatidylethanolamine containing epinephrine or arachidonic acid (AA) are key phospholipids involved in the induction of ferroptosis [21]. Additionally, other members of the ACSL family might substitute for ACSL4, playing a role similar to that of ACSL4 in mediating ferroptosis [43].

In summary, lipid peroxides are generated from intracellular polyunsaturated fatty acids as the foundational substrates. This occurs through a combination of enzymatic modifications and iron-mediated mechanisms. These lipid peroxides subsequently attack the cell membrane, resulting in ferroptosis. Lipid peroxidation constitutes a fundamental process in ferroptosis and represents a potential target for clinical intervention.

### Regulatory pathway of ferroptosis

In usual circumstances, cells possess intricate regulatory mechanisms to efficiently neutralize surplus peroxides and prevent ferroptosis-related reactions. Manipulation of these regulatory pathways can exert control over the onset of ferroptosis, holding promise for disease treatment. Consequently, ferroptosis regulation has emerged as a prominent research area in recent years. Among the identified ferroptosis-regulating mechanisms, the three primary pathways include the system Xc(−)-glutathione (GSH)-glutathione peroxidase 4 (GPX4) pathway, the nicotinamide adenine dinucleotide phosphate (NADPH)-ferroptosis suppressor protein 1 (FSP1)-coenzyme Q10 (CoQ10) pathway, and the GTP cyclohydrolase 1 (GCH1)-tetrahydrobiopterin (BH4) pathway. These pathways collectively orchestrate the cellular defense against ferroptosis [44].

#### The system Xc(−)-GSH-GPX4 pathway

The first regulatory pathway discovered and harnessed for inducing ferroptosis is the system Xc(−)-GSH-GPX4 pathway. This pathway relies on the catalytic activity of GPX4 and its cofactor GSH, which work in tandem to reduce lipid peroxides into harmless alcohols [45]. This enzymatic process is a crucial antioxidant mechanism within cells. Cysteine, the fundamental building block for GSH synthesis, is primarily supplied through the action of the system Xc- transporter protein on the cell membrane. System Xc- is a heterodimeric protein complex composed of solute carrier family 7 member 11 (SLC7A11/xCT) and solute carrier family 3 member 2 (SLC3A2). It facilitates the transport of cystine into the cell in a 1:1 ratio, which is then rapidly converted back into cysteine. Conducted by Dixon et al. in 2012, it was demonstrated that a small compound named erastin induced a unique form of cell death, subsequently termed ferroptosis [6]. Erastin’s mode of action centered on inhibiting the system Xc- transporter, thereby disrupting the cell’s ability to scavenge peroxides by reducing cellular cystine uptake. It was further observed that the tumor suppressor protein p53 downregulated the expression of SLC7A11, leading to ferroptosis via a similar mechanism [46, 47]. Additionally, the deletion of SLC7A11 selectively induced ferroptosis in pancreatic ductal adenocarcinoma cells driven by the KRAS proto-oncogene, effectively impeding tumor growth [48]. These findings illuminated the critical role of the system Xc- in ferroptosis regulation and its potential as a therapeutic target.

The synthesis of GSH plays a critical role in cellular ferroptosis. Activation of GSH synthase inhibitors can lead to ferroptosis [49–51]. Glutamate-cysteine ligase (GCL) is responsible for catalyzing the connection of cysteine to glutamate, a crucial step in the rate-limiting process of glutathione synthesis. The nuclear factor erythroid 2-related factor 2 (Nrf2) acts as a counterbalance to ferroptosis by promoting the expression of the GCL gene [52, 53]. Moreover, the expression of the multidrug resistance pump P-glycoprotein makes cells more susceptible to ferroptosis by pumping glutathione out of the cells [54]. This discovery reasonably explains why traditional drug resistance and ferroptosis sensitivity often appear at the same time and presents a novel approach for targeting drug-resistant bacteria or cancer cells. On the other hand, cells have alternative mechanisms to acquire cysteine, rendering them resistant to ferroptosis induced by compounds like erastin. Some cells can biosynthesize cysteine from methionine via the transsulfuration pathway. By blocking the transsulfuration pathway, cysteinyl-tRNA synthetase 1 (CARS1/CARS) facilitates erastin-induced ferroptosis [55]. In conclusion, reducing the cellular GSH concentration and diminishing the antioxidant capacity of cells, through means such as inhibiting system Xc- and other approaches, can induce ferroptosis.

Different from the effects of erastin, RSL3-induced ferroptosis doesn’t significantly alter intracellular GSH levels. However, it leads to a significant production of intracellular lipid peroxides. This difference indicates that RSL3 targets a protein, distinct from erastin, to modulate the accumulation of peroxides. In a proteomic study based on an erastin experiment, it was discovered that RSL3 skips the upstream system Xc- and covalently binds to GPX4, reducing its functionality [56]. This effect was further confirmed when researchers overexpressed GPX4 in colorectal cancer cells, resulting in the inhibition of ferroptosis induced by RSL3 [57]. GPX4 emerges as a pivotal factor in the regulation of ferroptosis. It’s important to note that GPX4 is a selenoprotein, with selenocysteine serving as its active component. Selenium has been shown to decrease the rate of ferroptosis when added to cells or administered to animals, including mouse models of brain hemorrhage. Consequently, selenium may influence the sensitivity of cells to ferroptosis [58–60]. Furthermore, apart from GPX4, other selenoproteins might also play a role in ferroptosis [34]. In conclusion, in the system Xc(−)-GSH-GPX4 pathway, ferroptosis can be induced by inhibiting the system Xc- or GPX4. This dual mechanism provides a potential therapeutic approach for related diseases by targeting ferroptosis.

#### The NADPH-FSP1-CoQ10 pathway

In 2016, Shimada K et al. identified FIN56 during an investigation of fifty-six caspase-independent lethal compounds. Their experiments revealed that FIN56 induces ferroptosis through a dual mechanism involving the depletion of GPX4 and CoQ10. This discovery unveiled a novel pathway for regulating ferroptosis [61]. CoQ10 plays a vital role as an antioxidant in vivo, capturing free radical intermediates and preventing lipid peroxidation. Consequently, the depletion of CoQ10 heightens cellular susceptibility to ferroptosis. Experimental evidence has shown that statins, which inhibit 3-hydroxy-3-methylglutaryl-coenzyme A (HMG-CoA) reductase activity, can also increase the rate of ferroptosis by depleting cellular CoQ10 [61]. Researchers investigating how cells prevent ferroptosis in the absence of GPX4 discovered the flavoprotein apoptosis-inducing factor mitochondria-associated 2 (AIFM2), subsequently renamed FSP1. Further research revealed that FSP1 counteracts lipid peroxides by catalyzing the regeneration of reduced-state CoQ10 using NAD(P)H. This finding suggests that FSP1 regulates ferroptosis through a distinct pathway running parallel to the system Xc(−)-GSH-GPX4 pathway, known as the NADPH-FSP1-CoQ10 pathway [62–64].

#### The GCH1-BH4 pathway

GCH1 serves as the rate-limiting enzyme in the synthesis of BH4, a crucial coenzyme involved in phenylalanine metabolism. BH4 also contributes to the production of CoQ10 in its reduced state, bolstering the cell’s capacity to neutralize lipid peroxides and inhibit ferroptosis. Furthermore, BH4 directly reduces the ferroptosis rate by blocking the peroxidation of specific lipids [65]. Consequently, modulation of the GCH1-BH4 pathway can independently intervene in the process of ferroptosis.

### The role of mitochondria in ferroptosis

A complex regulatory network involving multiple organelles collaborates to orchestrate ferroptosis, with mitochondria assuming particularly vital roles in this process. Experimental evidence has indicated a substantial increase in nonheme iron accumulation and lipid peroxidation specifically within mitochondria, rather than in the cytoplasm, during myocardial ferroptosis induced by Adriamycin [66]. While this outcome underscores the critical involvement of mitochondria in ferroptosis, the precise nature of their role remains unclear and warrants further investigation [51].

Research has shown that the depletion of mitochondria through parkin-mediated mitophagy effectively prevents ferroptosis induced by cysteine deprivation but doesn’t impact ferroptosis induced by GPX4 inhibition [67]. This observation suggests that mitochondria may promote ferroptosis when cysteine levels are deficient by influencing GSH metabolism. Further studies have highlighted the importance of mitochondrial glutamine degradation in initiating ferroptosis [26]. Two primary metabolic pathways of glutamine are the tricarboxylic acid (TCA) cycle and glutaminolysis [68]. The byproduct of glutamine catabolism, α-ketoglutarate (α-KG), and its downstream products within the TCA cycle are essential for the start of ferroptosis [26]. Moreover, in the absence of cysteine, glutamine catabolism enhances mitochondrial respiration and depletes GSH through GPX4, thereby promoting ferroptosis. Conversely, the prevention of ferroptosis induced by cysteine deficiency becomes possible when glutamine catabolism is suppressed [67]. This underscores the intricate relationship between mitochondrial function and ferroptosis regulation.

Mitochondrial lipids also play a significant role in inducing lipid peroxidation and subsequently ferroptosis. Knockdown of acyl CoA synthetase family member 2 (ACSF2) and citrate synthase (CS), both essential for mitochondrial lipid metabolism, has been shown to reverse erastin-induced ferroptosis [6]. Erastin targets the mitochondrial resident voltage-dependent anion channel 2/3 (VDAC2/3) located on the mitochondrial membrane. The interaction between VDAC2/3 and erastin impedes the entry of endogenous substrates and reduces the rate of NADH oxidation, leading to mitochondrial dysfunction and the release of oxidants, ultimately triggering cellular ferroptosis. Consequently, reducing the expression of VDAC2/3 can prevent ferroptosis induced by erastin [69, 70]. These findings underscore the close association between mitochondria and ferroptosis, making mitochondria a viable target for modulating ferroptosis.

### The ferroptosis propagation

In addition to killing individual cells, it has been discovered that ferroptosis initiates a cascade of ferroptosis triggers among cell populations in a wave-like fashion. This results in a distinctive spatiotemporal pattern of cell death, which has been seen in cells exposed to C’dots nanoparticles, which induce ferroptosis, as well as in the kidney tubules of mice treated with erastin [71, 72]. However, this wave-like action is mediated by specific ferroptosis subtypes that depend on the continual presence of iron and lipid peroxides. In various circumstances, the existence of non-random spatiotemporal patterns of ferroptosis has been statistically demonstrated by Riegman M et al. [73]. Ferroptosis induced by GPX4 inhibition is described as "single cell ferroptosis" and does not trigger a propagation among neighboring cells. In contrast, ferroptosis caused by glutathione inhibition (such as BSO and erastin) or cellular iron overload (like C’dots and FAC) can initiate ferroptosis in multiple cells and is characterized as "multiple cell ferroptosis." [73]. The failure of ferroptosis propagation following GPX4 inhibition may be associated with factors related to glutathione function or iron activity rather than GPX4 itself [54]. This distinction highlights the complexity of ferroptosis regulation and propagation within cell populations.

**Fig. 3:**
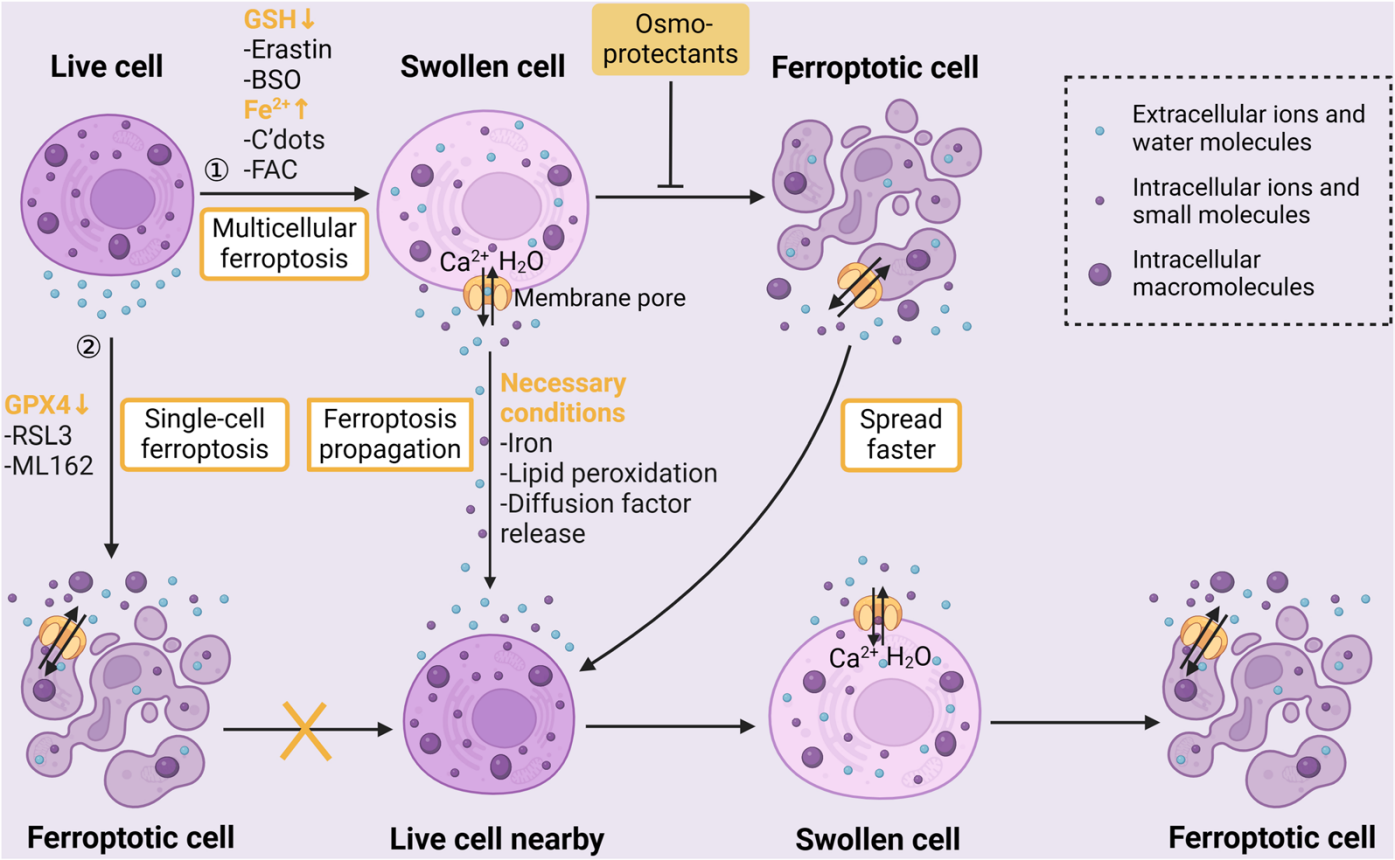
Mechanism of ferroptosis propagation between cells. This figure shows the inducers, conditions, processes, and changes in cell morphology and contents for the intercellular spread of ferroptosis, including single cell ferroptosis and multiple cell ferroptosis. (Created with BioRender.com.)

The complete process of ferroptosis, including cell lysis, might be necessary for its propagation. But in recent years, it has been found that it can also propagate in the cell population without cell rupture [73]. Ferroptotic cells undergo swelling and rounding before eventually rupturing. This swelling and rounding are caused by the formation of pores in the plasma membrane, which allow the influx of water molecules and ions from the external environment [74, 75]. What’s intriguing is that lipid peroxidation, a key aspect of ferroptosis, can alter the shape of lipid domains and sections of the plasma membrane [76, 77]. This suggests the possibility that lipids themselves, rather than pore-forming proteins, mediate the formation of plasma membrane pores. Cells can propagate ferroptosis before they rupture by releasing factors through these plasma membrane pores, and this process can be effectively prevented by osmoprotectants [75]. However, research has shown that when cells undergo complete lysis (rupture), ferroptosis propagation is accelerated [73]. This acceleration is likely due to the release of more diffusible components from the ruptured cells. Furthermore, arachidonic acid, a ferroptosis inducer, has been shown to cause significant cellular deformation in zebrafish larvae, indicating that ferroptosis can also propagate in vivo and lead to substantial tissue damage [78]. Therefore, further investigation into the cascade of events in ferroptosis propagation could have significant clinical implications. The mechanism of intercellular ferroptosis propagation is shown by a picture (Fig. 3).

## The role of ferroptosis in cancer

As a death mode first discovered in cancer [6], ferroptosis is closely related to the pathological process, metabolic state and microenvironment regulation of cancer. Therefore, we are here to elaborate the molecular mechanism related to ferroptosis in cancer.

### Ferroptosis is involved in the formation and regulation of cancer

Studies have found that some classic cancer-related factors and pathways can affect the formation and regulation of cancer by inducing or inhibiting ferroptosis [79], indicating that ferroptosis is widely involved in the pathological process of cancer.

#### TP53

TP53 gene encodes an important tumor suppressor P53, which is mutated or inactivated in about 50% of cancers [80], leading to the development of cancer. The typical and well-known anticancer effect of P53 is achieved by inducing cell cycle arrest, senescence or apoptosis of tumor cells [81–83]. However, in recent years, it has been found that P53 can also affect tumor growth by inducing ferroptosis, which is called atypical effect. P53 3KR, a mutant product of TP53 gene, has lost the ability to induce cell cycle arrest, senescence and apoptosis, but it can enhance the susceptibility of cells to ferroptosis [47, 84]. The P53 3KR knockin mice will not form spontaneous tumors, which proves the existence of ferroptosis-induced anti-tumor pathway of p53 [47]. As mentioned above, under cellular stress, P53 affects the ability of cells to remove excess lipid peroxides and promotes ferroptosis of tumor cells mainly by mediating the transcriptional inhibition of SLC7A11. For example, the activation of p53 by nutlin-3 will trigger ferroptosis of osteosarcoma U2OS cells [47]. P53 R273H and P53 R175H, two mutants of P53, cannot bind to DNA, but can still inhibit the expression of SLC7A11 by inhibiting the activity of other transcription factors, which indicates that p53 participates in an integrated transcription factor network to regulate ferroptosis [85]. In addition, P53 can also indirectly mediate ferroptosis through metabolic target genes such as SAT1 [86], FDXR [87] and GLS2 [26].

However, under basal or low ROS stress, P53 may in turn inhibit ferroptosis, indicating its bidirectional effect [88]. For example, the complex formed by dipeptidyl peptidase-4 (DPP4) and NOX1 can mediate plasma membrane lipid peroxidation. By binding and blocking the activity of DPP4, P53 can inhibit ferroptosis induced by erastin in human colorectal cancer cells [89]. Moreover, in fibrosarcoma cells, P53 can limit ferroptosis by inducing CDKN1A expression [90].

To sum up, TP53, as an important regulatory gene of both ferroptosis and tumor, usually promotes ferroptosis and inhibits tumor growth, and may has the opposite effect in specific cases. Eprenetapopt and coti-2, both aimed at reactivating p53, are currently being tested in clinical trials involving patients with acute myeloid leukemia (AML; NCT03931291) and various solid malignant tumors (NCT04383938 and NCT02433626).

#### RAS

RAS genes, such as HRAS, NRAS and KRAS, are the most commonly mutated oncogenes in cancer [91], and closely related to ferroptosis. As mentioned above, erastin and RSL3 can significantly induce ferroptosis in RAS mutant cancer cells [6, 57, 92]. This is because the mutant RAS signal may increase the concentration of intracellular free iron by regulating the expression of iron metabolism-related genes (such as FTH1 and TFRC), which in turn increase the sensitivity of cells to ferroptosis [27, 93]. Therefore, in recent years, anti-tumor drugs targeting RAS to induce ferroptosis have been developed one after another. For example, sotorasib and adagrasib, the inhibitors against KRAS-G12C mutant protein have been proved to have good activity in patients with non-small-cell lung cancer (NSCLC) and other solid tumors [94, 95]. However, the RAS mutation of tumor may also inhibit ferroptosis under certain circumstances. Ectopic expression of oncogenic RAS mutants (NRAS12V, KRAS12V and HRAS12V) was found to enhance the resistance of rhabdomyosarcoma RMS13 cells to oxidative stress and ferroptosis [96]. In addition, a sensitivity analysis of 177 cancer cell lines to common ferroptosis-induced small molecules showed that the mechanism of ferroptosis can be RAS-dependent or independent, indicating that RAS mutation is not a necessary condition for ferroptosis in tumors [97]. In a word, RAS shows its potential as an ferroptosis inducing target to resist tumors. Its different mutation characteristics have different responses to ferroptosis, which needs further exploration.

#### Other tumor-related factors

The tumor suppressor BAP1 encodes a nuclear deubiquitinating enzyme, which is in the form of a polycomb-repressive deubiquitinase (PR-DUB) complex to reduce the ubiquitination of histone 2A (H2A) in nucleosomes, so as to perform epigenetic regulation gene expression [98, 99]. Studies have shown that the anti-tumor effect of BAP1 is partly due to the ubiquitination of H2A on the promoter of SLC7A11, thus inhibiting the expression of SLC7A11 and inducing ferroptosis [100]. However, the germline mutation of BAP1 is widely found in many cancers and makes tumor cells lose the vulnerability to ferroptosis, which is considered as an important susceptibility factor of hereditary cancers [101–103].

As an important regulator of oxidative stress signaling, NFE2L2 can promote the formation, progress and drug resistance of tumors [104, 105]. It has been found that NFE2L2 can help cells to resist oxidative stress of ferroptosis by activating protective genes involved in iron metabolism (including SLC40A1, MT1G, HMOX1 and FTH1), GSH metabolism (including SLC7A11, GCLM and CHAC1) and ROS detoxification (including TXNRD1, AKNRD1, AKR1C1, etc.) [106].

Hypoxia promotes tumor development and drug resistance [107]. The expression of HIF, the main regulator of hypoxia, can promote fatty acid uptake by increasing the expression of fatty acid binding proteins 3 and 7, so as to avoid ferroptosis caused by lipid peroxidation in HT-1080 fibrosarcoma cells [108]. On the contrary, the activation of HIF can also lead to ferroptosis vulnerability of clear-cell carcinomas [109]. This shows that HIF seems to have a dual role in the modulation of ferroptosis in cancer cells.

The epithelial-to-mesenchymal transition (EMT) is the process by which epithelial cells lose their junctions and apical-basal polarity, and then increase the mobility of single cells and make the development of invasive phenotype possible [110]. It can lead to cancer spread and drug resistance [110]. Studies have shown that the high mesenchymal-like cell state in human cancer cell lines and organs is related to the selective vulnerability to ferroptosis [111]. Moreover, metadherin, a positive regulator of EMT, can promote ferroptosis in many cancer cell lines by inhibiting the expression of GPX4 and SLC3A2 [112]. In addition, EMT also destroys cadherin 1-mediated cell–cell contact that can prevent ferroptosis [113–115]. Therefore, it can be seen that the tumor-promoting effect of EMT is accompanied by ferroptosis susceptibility, which is expected to become a breakthrough in the treatment of tumors with EMT phenomenon.

### The role of ferroptosis in tumor microenvironment (TME)

The tumor microenvironment (TME) is composed of many different cellular and non-cellular components, which jointly drive tumor growth, invasion, metastasis and response to treatment. It makes cancer research change from a cancer-centered model to a model that regards TME as a whole [116, 117]. Immune cells, including T cells, macrophages, NK cells and so on, play a very important role in TME [118]. Moreover, a large number of studies have found that immune cells in TME have many overlaps with tumor cells in growth signals and metabolic characteristics, which further shows that they are closely interacted [119–122]. Therefore, we’ll ask two questions: does ferroptosis also occur in immune cells in TME when it’s induced in cancer cells and what is the interaction between immune cells and ferroptotic cancer cells.

#### Sensitivity of immune cells in TME to ferroptosis

T cells play an important role in anti-tumor immunity [123]. However, it was found that T cells lacking GPX4 will rapidly accumulate membrane lipid peroxides (LPO) after activation and lead to cell death, which will weaken their proliferation and anti-infection effects [124]. After that, this process was repeated in melanoma-related CD8^+^ T cells with the help of a high-throughput in vitro pharmacologic screening platform, and overexpression of GPX4 can effectively restore the anti-tumor immune effect of T cells [125]. Combined with the fact that cancer cells can promote the accumulation of reactive oxygen species in TME [126], it is reasonable to think that CD8^+^ T cells in TME are vulnerable to ferroptosis, and their susceptibility may be higher than that of T cells in physiological environment. This study also found that CD8^+^ T cells are even more sensitive to various ferroptosis inducers than some cancer cells (melanoma B16 cells) [125]. In contrast, Tregs in TME show a lower number of LPO, and are less prone to ferroptosis [125]. This might be because Tregs can rapidly induce the expression of GPX4 after being activated by TCR/CD28 co-stimulation [127]. However, targeted inhibition of GPX4 can still induce ferroptosis in Tregs to alleviate immunosuppression and exert antitumor effect [127].

In addition to T cells, other immune cells in TME will also respond to the induction of ferroptosis. It was found that M1 phenotype of tumor-associated macrophages (TAMs) is more resistant to ferroptosis caused by GPX4 deletion than M2 phenotype [128]. This might be because the high level of NO radical in M1 cells easily reacts with active intermediates produced by lipid free radicals and lipid peroxidation, and then replaces GPX4 to prevent ferroptosis [128, 129]. In addition, dendritic cells (DCs) have been proved to reduce the antigen processing ability due to ferroptosis when the lipid level increases [130–132]. Similarly, lipid peroxidation-associated oxidative stress caused by ferroptosis inhibits the glucose metabolism of NK cells to cause dysfunction, and the activation of Nrf2 antioxidant pathway may save them [133]. In short, not only tumor cells, but also immune cells in TME are susceptible to ferroptosis. The use of ferroptosis to treat tumors is a "double-edged sword", so efforts should be made to make the killing effect of tumors outweigh the damage to the immune system.

#### Interaction between ferroptotic cancer cells and immune cells in TME

Ferroptotic cancer cells and immune cells in TME can regulate each other. Firstly, ferroptosis can increase the immunogenicity of cancer cells. Studies have shown that cells undergoing early ferroptosis (within 1 h of treatment with the ferroptosis inducer RSL3) can induce dendritic cells to mature in vitro to kill fibrosarcoma cells by releasing damage-related molecular patterns (DAMPs) [134, 135]. Recently, a membrane oxidized phospholipid, 1-steaoryl-2–15-HpETE-sn-glycero-3-PE, has been found on the surface of ferroptotic cancer cells, which can guide macrophages to phagocytize [129]. But in some cases, ferroptotic cancer cells can also inhibit anti-tumor immunity. For example, the KRAS protein released from exosomes of ferroptotic tumor cells can be absorbed by TAMs. This, in turn, causes TAMs to switch to the M2 phenotype, thereby promoting the proliferation of pancreatic tumor [136].

Secondly, immune cells can also directly kill tumor cells through ferroptosis. Research shows that immunotherapy-activated CD8^+^ T cells can enhance ferroptosis-specific lipid peroxidation in tumor cells by downregulating the expression of SLC3A2 and SLC7A11, via the release of IFN-γ [137].

To sum up, ferroptosis can affect the whole TME, not just tumor cells, and it can also serve as a bridge between tumor cells and related immune cells. Therefore, ferroptosis is both an internal factor and an external factor of TME regulation.

## Progress of research on ferroptosis in sarcoma treatment

**Fig. 4:**
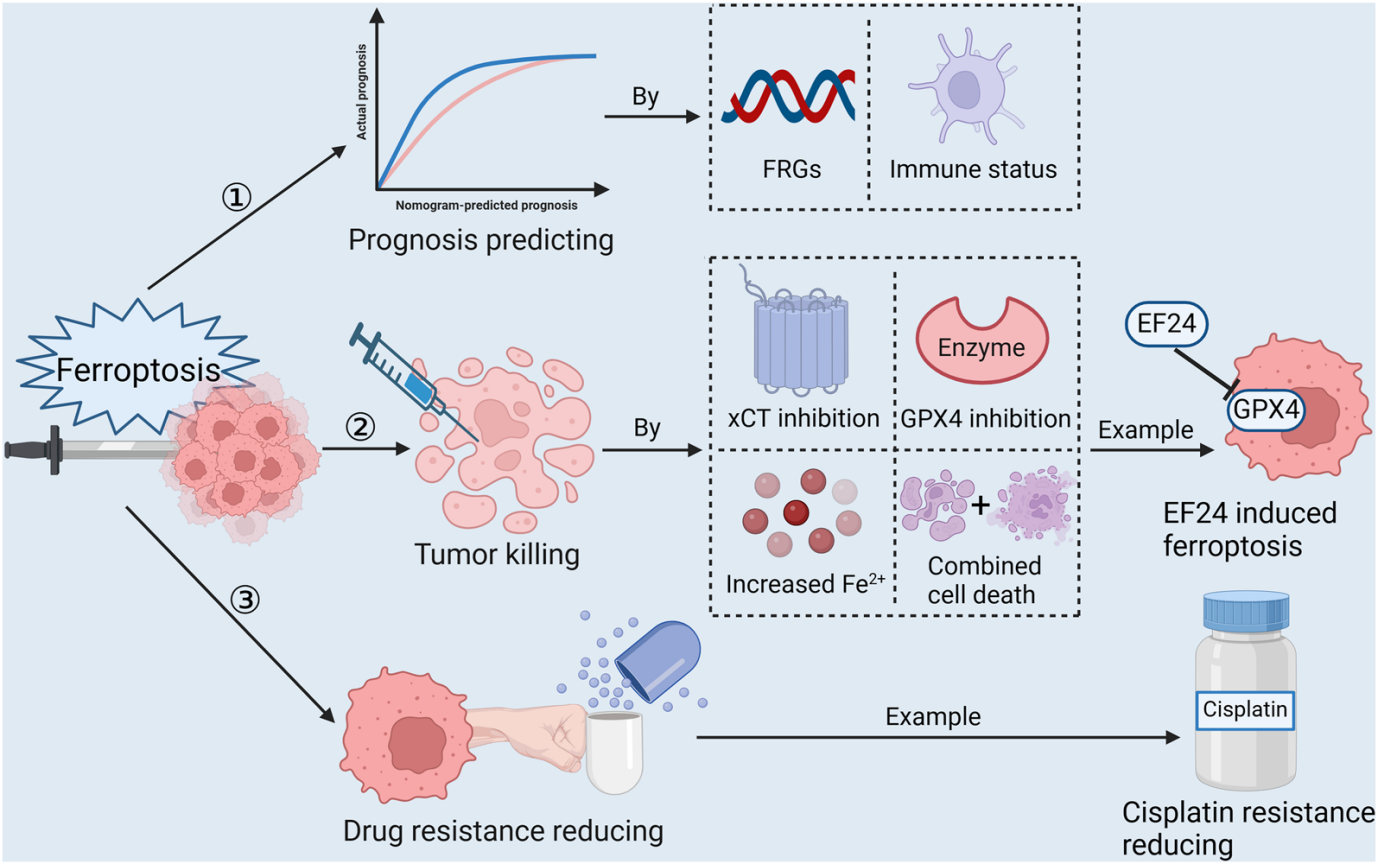
Pathways of ferroptosis for sarcoma treatment. This figure shows three common ways in which ferroptosis is applied to sarcoma treatment, including predicting prognosis, killing sarcomas, and reducing drug resistance, with clarification of mechanisms and examples. (Created with BioRender.com.)

The importance of conducting further clinical research on sarcoma treatment cannot be overstated, primarily due to the high mortality rates among patients, the limited range of effective therapeutic options available, and the persistent challenge of drug resistance in treating this condition. Ferroptosis, a recently explored form of programmed cell death, has shown promise in effectively restraining tumor growth, invasion, and progression [28]. Consequently, there is a growing body of research focused on exploring the diverse strategies through which ferroptosis can be harnessed as a potential treatment approach for sarcoma. (Fig. 4).

### Research progress on ferroptosis applied to directly kill sarcoma cells

The current standard treatment for sarcoma, which involves surgery along with radiation and chemotherapy, has limitations in terms of its effectiveness, especially in cases of metastatic tumors, where the median survival rate is only 15–20 months [138, 139]. Consequently, there is a pressing need for novel and more potent systemic therapies for sarcoma patients [140]. Ferroptosis has emerged as a potentially transformative mechanism for targeting and eliminating sarcoma cells. What’s particularly promising is that there is evidence to suggest that tumor cells, including sarcoma cells, may be more vulnerable to ferroptosis than normal cells, thanks to the correlation between the expression of cancer-related genes and ferroptosis-related genes (FRGs) [114, 141–143]. As a result, ferroptosis has garnered significant attention as a research focal point in the pursuit of more effective sarcoma treatments. In Tables 1 and 2, we’ve summarized the mechanisms, effects, and the cell models used in some common ferroptosis inducers and inhibitors, both for sarcoma cell-targeted and non-sarcoma cell-targeted treatments.

**Table 1:** Ferroptosis inducers targeting sarcoma cells and other cells

Compound	Mechanism	Effect	Cell models	Refs.
① Ferroptosis inducers in sarcoma				
5-aminolevulinic acid (ALA)	HMOX1 overexpression, iron and lipid peroxides overload	Fe^2+^ ↑, GPX4 ↓, ROS ↑, MDA ↑	SW872 (liposarcoma), MG63 (osteosarcoma)	[144]
ACXT-3102	SLC7A11 inhibitor	GSH ↓	SK-LMS-1, MG-63, HTB-93, etc (synovial sarcoma)	[145]
Bavachin	Transferrin receptor ↑, divalent metal transporter-1 ↑, ferritin light chain ↓, ferritin heavy chain ↓, p53 ↑, p-STAT3 ↓, SLC7A11 ↓, GPX4 ↓	Fe^2+^ ↑, GSH ↓, GPX4 ↓, ROS ↑, Malondialdehyde ↑, mitochondrial morphology alteration	MG63, HOS (osteosarcoma)	[146]
Buthionine-sulfoximine (BSO)	GCL inhibitor	GSH ↓	S4MH, F21 (rhabdomyosarcoma)	[147, 148]
β-Phenethyl isothiocyanate (PEITC)	TfR1 ↑, FPN, FTH1, DMT1 and IRP2 ↓, GSH/GSSG and GPX4 ↓	Fe^2+^ ↑, GSH ↓, GPX4 ↓, ROS ↑	MNNG/HOS, U-2 OS, MG-63, 143B, K7M2 (osteosarcoma)	[149, 150]
EF24	HMOX1 overexpression	Fe^2+^ ↑, GPX4 ↓, ROS ↑, MDA ↑	U2os, Saos-2 (osteosarcoma)	[11]
Erastin	SLC7A11 and VDAC2/3 inhibitor	GSH ↓, ROS ↑	HT1080; C2C12, RD, RH18, RH30, etc (various sarcomas)	[6, 92, 151, 152]
Ferric ammonium citrate (FAC)	Iron supplement	Fe^2+^ ↑	K7M2 (murine osteosarcoma)	[153]
Ferrous ammonium sulfate (FAS)	Iron supplement	Fe^2+^ ↑	K7M2 (murine osteosarcoma)	[153]
KDM4A	H3K9me3 demethylation in the promoter region of SLC7A11	GSH ↓	143 B, HOS (osteosarcoma)	[154]
MicroRNA-1287-5p	Bound to the 3'-untranslated region of GPX4	GPX4 ↓	Human osteosarcoma cells	[155]
Pure Artemisinin/*Artemisia annua L.* hydroalcoholic extract	Ferritin autophagy	Fe^2+^ ↑	D-17, OSCA-8, OSCA-40 (canine osteosarcoma)	[156, 157]
RSL3	GPX4 inhibitor	GPX4 ↓	BJ-TERT; HT1080; C2C12, RD, RH18, RH30, etc (various sarcomas)	[92, 97, 152]
Sorafenib	SLC7A11 inhibitor	GSH ↓	RH30, RD, RMS, etc. (rhabdomyosarcoma)	[147, 158]
Sulfasalazine (SAS)	SLC7A11 inhibitor	GSH ↓	K7M2 (murine osteosarcoma)	[153]
Theaflavin-3,3'-digallate (TF3)	Down-regulating FTH and GPX4, GSH consumption	Fe^2+^ ↑, GSH ↓, GPX4 ↓ ROS ↑, MDA ↑	MG63. HOS, hFOB1.19 (osteosarcoma)	[159]
Tirapazamine (under hypoxia)	SLC7A11 and GPX4 inhibitor; up-regulating p53	Fe^2+^ ↑, GSH ↓, GPX4 ↓	143B, U2OS, MNNG/Hos (osteosarcoma)	[160]
Ursolic acid	Ferritin autophagy	Fe^2+^ ↑	143 B, HOS (osteosarcoma)	[161]
Zoledronic acid	Up-regulating POR, down-regulating CoQ10, HMOX1 overexpression	ROS ↑, lipid peroxides ↑	Human osteosarcoma cells	[162, 163]
② Ferroptosis inducers in other cells				
BAY 11–7085	NFKBIA/IkBa inhibitor, HMOX1 overexpression	Fe^2+^ ↑, GPX4 ↓, ROS ↑, MDA ↑	MDA-MB-231, MCF-7, MDA-MB-468, SKBR3	[164]
BAY 87–2243	Mitochondrial complex I inhibitor	Mitochondrial membrane potential ↓, ROS ↑	G361, SK-MEL-28	[165]
Cyst(e)inase	Cyst(e)ine consumption	GSH ↓	AsPC-1, PANC-1, BxPC-3, S2-013; PCa cells, FVB/N mice	[48, 166]
FeCl2	Iron supplement	Fe^2+^ ↑	OHSCs	[167]
FIN56	GPX4 consumption and depleting CoQ10 via the mevalonate pathway	GPX4 ↓, CoQ10 ↓, ROS ↑	BJeLR, HT-1080, MEFs, PACN1	[61, 168]
FINO2	Indirect inhibitor of GPX4 and direct oxidant of iron	Fe^2+^ ↑, GPX4 ↓	HT-1080	[169]
Glutamate	SLC7A11 inhibitor	GSH ↓	HT1080, PC12	[170]
Hemin	HMOX1 overexpression and iron supplement	Fe^2+^ ↑, GPX4 ↓, ROS ↑, MDA ↑	IMR-32, SK-N-SH; male Swiss albino mice	[23, 171]
Hemoglobin	Iron supplement and ROS production	Fe^2+^ ↑, ROS ↑, MDA ↑	OHSCs	[167]
(NH4)2Fe(SO4)2	Iron supplement	Fe^2+^ ↑	IMR-32	[171]
Piperazine erastin	SLC7A11 inhibitor	GSH ↓	HT-1080, BJeLR	[6, 97]
Statins (fluvastatin, lovastatin, simvastatin)	HMG-CoA reductase inhibitor and GPX4 biosynthesis suppression	GPX4 ↓, CoQ10 ↓, ROS ↑	HT-1080, HCC4006	[61, 111]

**Table 2:** Ferroptosis inhibitors targeting sarcoma cells and other cells

Compound	Mechanism	Effect	Cell model	Refs.
① Ferroptosis inhibitors in sarcoma				
Bathophenanthrolined-isulfonic acid (BPS)	Iron chelator	Fe^2+^ ↓	C2C12, U57810 (rhabdomyosarcoma)	[92]
Bisindolylmaleimide I and Gö6976	Protein kinase C inhibitor	Lipid peroxides ↓	RD, RH18, RH30	[151]
Deferoxamine (DFO)	Iron chelator	Fe^2+^ ↓	MG63, HOS (Osteosarcoma); HT1080 (fibrosarcoma)	[6, 146]
Diphenyleneiodonium chloride (DPI) and GKT137831	NOX inhibitor	NOX-mediated lipid peroxidation ↓, ROS ↓	RD, RH18, RH30	[151]
Fanconi anemia complementation group D2 (FANCD2)	JAK2/STAT3 pathway inhibitor, FTH1 ↑, GPX4 ↑, COX2 ↓, LIP ↓	GPX4 ↑, Fe^2+^ ↓, ROS ↓	MG-63, U2OS, hFOB1.19 (Osteosarcoma)	[172]
Ferrostatin-1	Radical-trapping antioxidants	ROS ↓, lipid peroxides ↓	HT1080, MG63, HOS, C2C12, RD, RH18, RH30, HEK-29, HT22, etc (various sarcomas)	[6, 92, 146, 151, 173]
Liproxstatin-1	Radical-trapping antioxidants	ROS ↓, lipid peroxides ↓	HT1080, MG63, HOS, HEK-29, HT22 (various sarcomas)	[6, 146, 173]
LncRNA- SNHG14	Down-regulating miR-206	GSH ↑	NR-SJSA1 (nutlin3a-resistant osteosarcoma)	[174]
Mitochondrial NADP + -dependent isocitrate dehydrogenase (IDH2)	NADPH production	GSH ↑	HT1080 (fibrosarcoma); Hepa1-6 (hepatoma)	[175]
N-acetyl cysteine (NAC), Glutathione (GSH)	Antioxidant, GSH supplement	GSH ↑, ROS ↓	HT1080, C2C12, U57810 (rhabdomyosarcoma)	[6, 92, 97]
Pifithrin-α	p53 inhibitor	GSH ↑	MG63, HOS (Osteosarcoma)	[146]
Vitamin E and tocopherols	Antioxidant, LOX inhibitor	ROS ↓, lipid peroxides ↓	MG63, HOS (Osteosarcoma), Pfa1	[21, 146]
ZnPPIX	Down-regulating HMOX1	Fe^2+^ ↓, GPX4 ↑, ROS ↓, MDA ↓	SW872 (liposarcoma), MG63 (osteosarcoma)	[144]
② Ferroptosis inhibitors in other cells				
1-methyl tryptophan	Indoleamine 2, 3-dioxygenase (IDO) inhibitor, up-regulating SLC7A11, reduction of nitrative stress	GSH ↑	LO2	[176]
AA-861	5-LOX inhibitor	ROS ↓, lipid peroxides ↓	HEK-293 T, G401	[177, 178]
Baicalein	12/15-LOX inhibitor	ROS ↓, lipid peroxides ↓	PANC1, BxPc3; Jurkat, Molt-4	[179, 180]
Butylated hydroxyanisole and butylated hydroxytoluene	Antioxidant	ROS ↓, lipid peroxides ↓	HT1080; male C57BL/6 J mice	[97, 181]
β-mercaptoethanol (2ME)	Reduction of cystine to cysteine	GSH ↑	BMDMΦ and OT-1 CD8^+^ T cells	[6, 182]
CoQ10, idebenone	Antioxidant	ROS ↓, lipid peroxides ↓	U-2 OS, NCI-H460, NCI-H2291, NCI-H1703, NCI-H446, HT1080	[62, 63]
Dopamine	Improvement of stability of GPX4	GPX4 ↑	HEK293, PANC1, HEY, MEF	[183]
Selenium	Active group of GPX4	GPX4 ↑	HT-1080, MEFs	[58]
Zileuton	5-LOX inhibitor	ROS ↓, lipid peroxides ↓	Pfa1, HT22	[21, 184]

#### Ferroptosis in the treatment of osteosarcoma

Certain drugs, such as bavachin [146], tirapazamine [160], and sulfasalazine [153, 185], have demonstrated the ability to induce ferroptosis in osteosarcoma cells by inhibiting the expression of the system Xc- component SLC7A11. In particular, bavachin has proven effective in inhibiting the growth of MG63 and HOS osteosarcoma cell lines. This inhibitory effect of bavachin on osteosarcoma cell growth can be reversed by ferroptosis inhibitors like ferrostatin-1 and liproxtin-1, iron chelators like desferrioxamine, and antioxidants like Vitamin E [146]. This suggests that bavachin induces cell death in osteosarcoma cells through ferroptosis. Further investigation into the mechanism revealed that bavachin increases the expression of transferrin receptor and divalent metal transporter-1 while decreasing the expression of ferritin light chain and ferritin heavy chain in osteosarcoma cells. This leads to an increase in intracellular ferrous iron content, making the cells more susceptible to ferroptosis. Additionally, bavachin upregulates the expression of p53 by downregulating phosphorylated signal transducer and activator of transcription 3 (p-STAT3). Then, p53 downregulates SLC7A11 and GPX4 expression, contributing to the accumulation of intracellular ROS and MDA. This finding highlights the importance of the STAT3/p53/SLC7A11 axis as a key pathway involved in ferroptosis induced by bavachin [146]. Conversely, the histone demethylase KDM4A has been found to increase SLC7A11 expression and inhibit ferroptosis in osteosarcoma cells by controlling the demethylation of H3K9me3 at the SLC7A11 promoter region [154]. Therefore, KDM4A is a potential therapeutic target for the treatment of osteosarcoma.

In addition to targeting the membrane receptor system Xc-, increasing the intracellular iron concentration has also been explored as a strategy to induce ferroptosis in sarcoma cells for potential treatment. Several studies have investigated the effects of iron supplementation on sarcoma cells. For instance, iron supplementation with compounds like ferric ammonium citrate (FAC) or ferrous ammonium sulfate (FAS) has been found to exacerbate ferroptosis induced by treatments in sarcoma cells, such as intensifying SAS-induced ferroptosis in K7M2 osteosarcoma cells [153]. This suggests that increasing intracellular iron levels can enhance ferroptosis in sarcoma cells. Another intriguing finding is related to ferritin autophagy, a process that involves the degradation of ferritin, which can increase the intracellular concentration of unstable iron. This mechanism appears to be critical for inducing ferroptosis in canine osteosarcoma cell lines when treated with a hydroalcoholic Artemisia annua extract [156, 157]. Furthermore, compounds like phenethyl isothiocyanate (PEITC), derived from cruciferous vegetables and available in plant extracts, have been shown to induce multiple forms of cell death, primarily ferroptosis, apoptosis, and autophagy, in osteosarcoma cells. This is achieved through mechanisms that include increasing active iron, depleting GSH, producing ROS, and activating the MAPK signaling pathway [149, 150]. However, it’s important to note that excessive PEITC intake can potentially affect normal cells due to expanded tissue distribution resulting from metabolic saturation [150, 186]. Additionally, EF24, a synthetic analog of curcumin, triggers ferroptosis by upregulating heme oxygenase 1 (HMOX1). This upregulation increases the Fe^2+^ concentration by breaking down heme and inhibiting GPX4 expression. EF24 is considered a promising candidate for treating HMOX1-positive osteosarcoma [11]. These studies demonstrate the potential of Chinese patent medicines in inducing sarcoma ferroptosis.

In vitro cell experiments showed that high concentrations of 5-aminolevulinic acid similarly induced ferroptosis in human sarcoma cells by overexpressing HMOX1 in the dark, suggesting new possibilities for the application of this drug [144]. Furthermore, zoledronic acid has exhibited multifaceted effects in promoting ferroptosis in osteosarcoma cells. It not only upregulates HMOX1 protein expression but also significantly reduces the levels of the antioxidant CoQ10. Additionally, zoledronic acid increases the expression of POR, an enzyme required for lipid peroxidation [162, 163]. These combined actions enhance the propensity for ferroptosis in osteosarcoma cells. Factors that can regulate ferroptosis in sarcoma cells by affecting both the Fe^2+^ concentration and GPX4 expression also include the fanconi anemia complementation group D2 (FANCD2) [172] and theaflavin-3,3′-digallate [159].

The latest approach to cancer treatment in recent years can be combined with ferroptosis to increase treatment efficacy. MicroRNAs (miRNAs) have emerged as promising candidates for personalized cancer treatment and appear to be involved in ferroptosis [187–190]. For instance, miR-1287-5p, which is downregulated in human osteosarcoma, exhibits upregulation in response to ferroptotic stimulation [155]. Elevated miR-1287-5p levels directly target the 3′-untranslated region of GPX4, inhibiting its activity and promoting ferroptosis in osteosarcoma cells. Additionally, miR-1287-5p mimics significantly heighten the sensitivity of human osteosarcoma cells to cisplatin chemotherapy [155]. Exosome-mediated miR-144-3p is another microRNA with a role in inhibiting osteosarcoma development. It regulates ZEB1 expression, thereby promoting ferroptosis [191]. Moreover, the long non-coding RNA (lncRNA) SNHG14 affects SLC7A11 activity and prevents ferroptosis by targeting and downregulating miR-206 expression in nutlin3a-resistant osteosarcoma cell lines [174]. Photodynamic therapy (PDT), which is a promising approach for various cancers, has been investigated in human osteosarcoma cells [192, 193]. Specifically, pyropheophorbide-α methyl ester-mediated PDT (MPPa-PDT) induces apoptosis while increasing unphosphorylated Yes-associated protein (YAP) levels, which in turn initiate Hippo pathway to inhibit apoptosis [194–196]. YAP knockdown enhances the sensitivity of human osteosarcoma cells to MPPa-PDT, increasing apoptosis rates and reducing drug resistance when administered in combination with erastin, an inducer of ferroptosis [196]. When utilized alongside homologous-sequence-targeting nanoparticles, PDT can further enhance apoptosis and ferroptosis rates in osteosarcoma cells [197].

#### Ferroptosis applied to the treatment of other sarcomas

Rhabdomyosarcoma (RMS) cells have been reported to be susceptible to oxidative stress, and the mechanism of increasing GSH to increase antioxidant defense makes these cells more vulnerable to GSH depletion [198]. As a result, ferroptosis may be applied as a new RMS treatment, especially for refractory RMS. Recent studies have demonstrated that erastin and RSL3 induce ferroptosis in rapidly proliferating myogenic cells via the extracellular signal-regulated kinase (ERK) pathway. When combined with chemotherapeutic agents like adriamycin and actinomycin D, these compounds effectively inhibit all RMS cell lines [92]. Furthermore, sorafenib, which targets system Xc-, and buthionine-sulfonylimine, an inhibitor of GSH biosynthesis, have demonstrated the ability to hinder RMS cell line growth [147, 148, 158]. Protein Kinase C (PKC) and NADPH Oxidase (NOX) are also involved in the regulation of ferroptosis in RMS cells [151].

Ewing sarcoma (ES) is one of the most common malignant tumors in children, with a high degree of malignancy and limited treatment options [199]. Therefore, it is extremely urgent to identify novel potential therapeutic targets for ES and put them into use in clinical settings. Studies have shown that aurora kinase A (AURKA) is significantly up-regulated in ES, and its expression level is significantly related to the short overall survival and event-free survival of patients with ES [200, 201]. AURKA inhibition can trigger the apoptosis and ferroptosis of ES cells through the NPM1/Yes1 associated transcriptional regulator (YAP1) axis. Subsequently, this study identified an AURKA inhibitor TCS7010, which has the killing effect on ES cells, through the high-throughput screening of a small molecular pharmacy library [201]. In addition, cytosolic carbonic anhydrase (CA) may also be a potential target for ES therapy, and CA inhibitors can induce ferroptosis through Inhibition of AKT/FTH1 signaling in ES Cells [202].

Expanding on the potential of ferroptosis as a treatment strategy for various types of sarcomas, Kim H and colleagues found that the deletion of isocitrate dehydrogenase (IDH) increased the sensitivity of human HT1080 fibrosarcoma cells to ferroptosis induction when cultured in vitro [175]. In the context of synovial sarcoma, which is characterized by a deficiency in malic enzyme 1, these sarcoma cells exhibited heightened susceptibility to ferroptosis triggered by ACXT-3102 [145]. SHARPIN, an activator of NF-kappaB, can also induce the ferroptosis of synovial sarcoma cells, and the PGC1α/NRF2/SLC7A11 axis and BNIP3L/NIX-mediated mitophagy is involved in its downstream regulation [203]. Moreover, in the case of uterine carcinosarcoma, knocking out the ferroptosis-related gene named heat shock factor 1 (HSF1) increased the sensitivity of tumor cells to treatment with adriamycin or gemcitabine, suggesting a potential combination therapy approach [204]. These findings underscore the versatility of ferroptosis-based treatments across different types of sarcomas.

The utilization of ferroptosis as a means to eliminate tumor cells represents an innovative approach to sarcoma treatment. Within this domain, the objectives moving forward encompass the discovery of novel ferroptosis-inducing agents and intervention targets. Furthermore, efforts are aimed at enhancing the efficacy of tumor eradication and refining drug therapies through rigorous clinical trials. This promising avenue of research holds the potential to revolutionize the treatment landscape for sarcomas.

### Targeting ferroptosis in immunotherapy to indirectly resist sarcoma

Immunotherapy for sarcoma has been studied for many years and achieved good results, which is an important supplement to chemotherapy [205, 206]. As mentioned above, ferroptosis can affect can affect tumor-related immune effects in TME. (chapter " The role of ferroptosis in TME "). Therefore, it is hopeful to enhance the immunotherapy effect of sarcoma by inducing ferroptosis, which can be achieved by two ways: inducing ferroptosis in sarcoma cells to enhance its immunogenicity or regulating ferroptosis in immune cells to enhance the anti-sarcoma immune effect. Efimova I et al. confirmed for the first time that ferroptosis is immunogenic in vivo and in vitro [135]. They found that early (rather than late) ferroptotic cells can promote the phenotypic maturation of bone marrow-derived dendritic cells (BMDCs) and elicit a vaccination-like effect in immune-competent mice but not in Rag-2-/-mice through the co-culture of ferroptotic mice fibrosarcoma MCA205 cells with immune cells in vitro and the preventive ferroptosis tumor vaccination inside the mice [135]. This indicates that the mechanism of ferroptosis-mediated immunogenicity is closely regulated by the adaptive immune system and is time-dependent. In theory, this method is expected to effectively reverse the treatment dilemma of patients with immune desert sarcoma. Some commonly used targeted therapies (such as sorafenib), chemotherapy (such as cisplatin) and radiotherapy for sarcoma are also ferroptosis inducers, which are helpful to enhance the immunogenicity of sarcoma cells [170, 207], reflecting the synergy between immunotherapy for sarcoma and other treatments.

Because immune cells are also vulnerable to ferroptosis (chapter “The role of ferroptosis in TME”), tumor immunotherapy can be carried out by regulating ferroptosis of immune cells. Common ideas include that ferroptotic stress inducing TAMs to repolarize from M2 type to M1 type [128, 208–210], inducing ferroptosis of Tregs to reduce negative immune effect [127], targeting system Xc- to alleviate cystine deprivation in TME mediated by myeloid-derived suppressor cells (MDSCs) to promote T cell survival [211] and so on. Recently, Yu K et al. designed a biomimetic hybrid cell membrane camouflaged by poly (lactic-co-glycolic) acid (PLGA)-loaded Fe_3_O_4_ and DHJS (a probe for ROS generation) to induce ferroptosis in osteosarcoma cells, and successfully mediated macrophage M1 polarization as well as the infiltration of CD8^+^ T cells and dendritic cells in tumors [212]. However, in sarcomas with different immunophenotypes, the effects of ferroptosis on tumor immunity may be different and different kinds of ferroptosis inducers will also have different effects on immune cells and tumor cells [121]. These factors determine the balance between tumor killing effect and immune system damage. Therefore, selecting the appropriate ferroptosis inducer for specific sarcoma is the most critical step for curative effect, and it is also a gap to be further explored.

### Ferroptosis reduces drug resistance of sarcoma cells to chemotherapeutic agents

Despite the progress made in increasing the 5-year survival rate of sarcoma patients through conventional surgical treatment and postoperative neoadjuvant chemotherapy, chemotherapy resistance remains a significant obstacle to improving patient outcomes [213–215]. Cisplatin, a highly potent and commonly used chemotherapeutic agent for solid tumors, exerts its anti-tumor effects by triggering both apoptosis and ferroptosis [216, 217]. Nevertheless, tumor cells can develop resistance to cisplatin by engaging mechanisms that regulate autophagy and enhance the expression of antioxidant enzymes [218–220]. While previous studies have largely focused on reactivating proapoptotic pathways to enhance the sensitivity of sarcoma cells to cisplatin [221–223], this approach has not proven to be particularly effective.

Drug-resistant cancer cells, particularly those reliant on the GPX4 antioxidant system, are susceptible to ferroptosis induction [111, 224]. Consequently, combining ferroptosis inducers represents a novel approach to combat sarcoma resistance to chemotherapeutic agents like cisplatin. For instance, the combination of the ferroptosis agonist erastin with cisplatin has demonstrated a significant synergistic effect against A549/HCT116 tumor cells [10]. Additionally, both erastin and STAT3 inhibitors have been effective in reactivating ferroptosis in drug-resistant tumor cells, rendering them more susceptible to cisplatin [220]. Furthermore, research has highlighted the potential of the plant extract ursolic acid (UA) as an adjunct to cisplatin treatment for sarcoma [161]. UA promotes tumor cell apoptosis, inhibits metastasis [225], and, in the presence of cisplatin, activates ferritin autophagy and degradation. This leads to increased free iron levels, lipid peroxide accumulation, and ferroptosis induction, underlining the close relationship between autophagy and ferroptosis in cancer cell death [161, 226]. Moreover, with the assistance of nanomaterial technology, anti-Her2 affibody-decorated arsenene nanosheets have proven effective in depleting intracellular GSH and inhibiting GPX4 activity, thus inducing ferroptosis and overcoming cisplatin resistance [227].

In conclusion, ferroptosis holds significant promise for overcoming the resistance of sarcoma cells to chemotherapeutic agents, particularly cisplatin. The synergistic combination of ferroptosis inducers with conventional chemotherapeutic agents represents a potentially transformative approach in the treatment of sarcoma.

### Predictive value of ferroptosis-related genes expression in sarcoma cells

Multiple molecular networks play critical roles in the regulation and understanding of ferroptosis. Exploring these molecular mechanisms can offer valuable insights into the potential clinical applications of ferroptosis in disease treatment. A growing body of research has identified and extensively studied ferroptosis-related genes (FRGs) across various fields [228, 229]. The expression of FRGs has been strongly associated with the development of several types of cancer, including hepatocellular carcinoma [230], glioma [231], esophageal adenocarcinoma [232], and lung adenocarcinoma [233]. Additionally, researchers have explored the use of FRGs in predicting the prognosis of sarcoma patients (Table 3).

**Table 3:** Summary of prognostic models for sarcomas based on FRGs

**Time**	**Target**	**Screening method**	**Hub genes for Prognostic model**	**Efficiency verification**	**Refs.**
2021	Soft tissue sarcoma	Univariate Cox → LASSO → Multivariate Cox	MUC1, GSS, HELLS, RPL8, ALOX15B, NOX5, CD44, ISCU, NCOA4, RGS4, SETD1B, GCLM	① GEO database ② ROC curve ③ K-M survival analysis	[234]
2021	Osteosarcoma	Univariate Cox → LASSO → Multivariate Cox	G6PD, PEBP1, PGD, DPP4, SLC39A8, SOCS1, ATG7, MYC, ALOX15B, CBS, EGLN1, MUC1	① GEO database ② Time-dependent ROC	[9]
2022	Soft tissue sarcoma	Log-rank → Wilcoxon rank sum → Univariate Cox → Multivariate Cox	EPAS1, STMN1, CXCL2, NQO1, HELLS, IL6	① GEO database ② Time-dependent ROC	[235]
2022	Uterine carcinosarcoma	Univariate Cox → LASSO	PGD, HSF1, ISCU, PLIN2, GPT2	① GEO database ② ROC curve	[204]
2022	Sarcoma	Univariate Cox → LASSO	SLC7A11, FANCD2, CISD1, ATP3MC3	① ROC curve ② K-M survival analysis	[236]
2022	Osteosarcoma and Chemotherapy resistance	Log-rank → Univariate Cox → Multivariate Cox	CBS, COCS1, EGFR	① GEO database ② Time-dependent ROC	[237]
2022	Osteosarcoma	Univariate Cox → LASSO → Multivariate Cox	PGD, G6PD, ACSF2, MT1G, FADS2, CBS	① ROC curve ② K-M survival analysis	[238]
2022	Ewing Sarcoma	Univariate Cox → Random survival forest algorithm → Multivariate Cox	AURKA, RGS4, RIPK1	① GEO database ② ROC curve ③ K-M survival analysis	[239]
2022	Osteosarcoma	Univariate Cox → LASSO	TP53, HMOX1, SLC7A11, HRAS, VEGFA, TXNRD1, CBS, G6PD	① GEO database ② ROC curve	[240]
2022	Osteosarcoma	Univariate Cox → LASSO → Multivariate Cox	LRRC1, ACO2, CTNNBIP1 (FRG subclusters)	① ROC curve ② K-M survival analysis ③ Calibration curves	[241]
2022	Osteosarcoma	WGCNA → LASSO → Multivariate Cox	COL5A2, HOXB4, UNC5B	① GEO database ② ROC curve ③ K-M survival analysis	[242]
2022	Osteosarcoma	Univariate Cox → LASSO → Multivariate Cox	ACSL4, HMOX1, GPX4, PRNP, ATG7	① TARGET and GEO database ② K-M survival analysis	[243]
2023	Osteosarcoma	Univariate Cox → LASSO → Multivariate Cox	ACSL5, ATF4, CBS, CDO1, SCD, SLC3A2	① GEO database ② ROC curve ③ Subgroup analysis	[244]
2023	Osteosarcoma	Univariate Cox → LASSO	MUC1, MAP3K5, LURAP1L, HMOX1, BNIP3	① GEO database ② ROC curve ③ Univariate and multivariate Cox	[245]

The first prognostic model for soft tissue sarcoma (STS) based on FRGs was developed by Huang W and colleagues [234]. They utilized RNA sequencing profile and employed various analytical techniques such as Cox regression analysis and LASSO analysis to identify 12 FRGs that are closely linked to the prognosis of STS. These FRGs were then integrated with clinical variables to construct a nomogram, which serves as a predictive tool for assessing the prognosis of STS patients. Furthermore, the independence and validity of these prognostic signals, along with the expression levels of key prognostic genes, were thoroughly validated in their study [234].

Subsequent to these developments, more researchers have endeavored to create prognostic models for sarcoma based on FRGs. For example, Lei T and his collaborators devised an innovative prognostic model specific to osteosarcoma [9]. Similarly, a prognostic model for Ewing sarcoma was successfully established, pinpointing three crucial genes, AURKA, RGS4, and RIPK1, that are intimately linked to disease prognosis [239]. As research efforts have expanded, an increasing number of key FRGs associated with sarcoma growth have been both identified and corroborated (Table 3 above). Furthermore, FRGs can serve as biomarkers to facilitate the screening of chemotherapeutic agents, thus aiding in the formulation of personalized chemotherapy strategies tailored to individual sarcoma patients [236, 239]. This same research strategy can also be applied to forecast tumor prognosis using ferroptosis-associated lncRNA genes [246].

GO and KEGG enrichment analyses have uncovered that FRGs are significantly enriched in pathways related to cancer and the immune system. Correlation analyses of key FRGs with immune checkpoint genes (ICGs) have revealed positive associations between the expression levels of CXCL2 and IL6, which are proteins encoded by FRGs, and the expression of immune factors [235]. RIPK1, a key FRG, has also been shown to be part of the same protein interaction network as immune checkpoints like PD-1 [239]. Furthermore, prognostic analyses based on FRGs have demonstrated that different risk groups exhibit varying patterns of immune cell infiltration, and individuals with more active immune cell involvement tend to have a better prognosis [9, 235, 237]. These findings suggest that FRG-encoded proteins can influence tumor prognosis by modulating the immune system, and further support the relationship between ferroptosis and tumor immune system.

## Challenges and prospects

Indeed, while the potential of ferroptosis as a therapeutic approach in sarcoma treatment is promising, there are several critical issues that need to be addressed before its widespread clinical application.

### Research perspectives on ferroptosis

Ferroptosis is a novel form of programmed cell death characterized by iron dependence and lipid peroxide accumulation, which differs from apoptosis, necrosis, and cellular autophagy. The activation of p53, a critical regulator of apoptosis, has been found to play a role in ferroptosis regulation. p53 can inhibit ferroptosis either by downregulating SLC7A11 expression or through the p53-p21 axis, indicating a connection between apoptosis and ferroptosis [46, 47, 90]. NCOA4-mediated ferritin autophagy can increase intracellular unstable iron levels, inducing cellular ferroptosis [30, 36, 247]. Interestingly, the ferroptosis inducer erastin can also promote ferritin autophagy, suggesting potential synergies between these two forms of cell death. Even whether ferroptosis is triggered by autophagy is a topic of debate in the scientific community, and further research is needed to clarify the relationship between these cell death modalities. Additionally, given the close ties between ferroptosis, the immune system, and lncRNA, there is potential for integrating these factors into a comprehensive regulatory network to better modulate cellular states and identify therapeutic targets.

Ferroptosis’s reliance on intracellular free iron is a defining feature. However, recent findings have revealed that copper, another crucial transition metal, can induce redox metabolism changes in cells similar to those seen with erastin-induced ferroptosis, by depleting GSH [248, 249]. Therefore, other metal ions, in addition to iron ions, have showed the potential to induce ferroptosis under specific conditions. This discovery raises questions about whether iron is necessary for ferroptosis and whether other metal ions or substances can disrupt intracellular redox balance and trigger ferroptosis. The emergence of cuproptosis underscores the potential for metal ions to influence cellular metabolism and cell death [250–253]. While cuproptosis and ferroptosis are currently understood to operate through distinct mechanisms, future research may uncover synergistic effects between them.

There is still much to explore in understanding the underlying mechanisms of ferroptosis, and further refinement of its description is necessary to support its clinical applications based on more comprehensive theories.

### Clinical application and challenges of ferroptosis in the treatment of sarcoma

As previously mentioned, ferroptosis can currently be applied to sarcoma treatment through three primary approaches. The first and most common potential clinical application involves directly inducing ferroptosis in sarcoma cells, leading to their demise. Several ferroptosis inducers have been identified that function by depleting intracellular GSH, inactivating GPX4, activating the mevalonate pathway, and increasing intracellular lipid peroxidation and iron content [18, 171, 254]. In this context, FDA-approved drugs like sorafenib and octreotide have demonstrated their ability to induce ferroptosis in refractory cancers [18, 255]. Small-molecule drugs and proprietary Chinese medicines can also serve similar roles [11, 146, 160].The therapeutic significance of ferroptosis therapy is underscored by the observation that certain cancers inherently possess characteristics that make them sensitive to this form of cell death. For instance, rhabdomyosarcoma cells are susceptible to oxidative stress [151], while synovial sarcoma cells exhibit recurrent malic enzyme 1 expression deficiency [145], rendering them less tolerant to lipid peroxide accumulation and ferroptosis. Nevertheless, it’s important to note that not all cancer cells respond to ferroptosis inducers, and even well-characterized RAS-mutant cancer cell lines may not exhibit susceptibility [10, 45, 97]. Consequently, a key pending strategy is how to enhance tumor cell sensitivity to ferroptosis through epigenome editing, potentially paving the way for clinical applications of ferroptosis-based therapy. Alternatively, therapeutic effectiveness can be increased by exploiting tumor-specific characteristics. For instance, osteosarcoma cells display high expression of HMOX1, so EF24, which promotes HMOX1 expression, can be employed to expedite ferroptosis in these cells [11].

In addition to enhancing sensitivity, the targeting and absorption rate by cancer cells are factors that limit the clinical efficacy of ferroptosis-inducing treatments. Erastin, as the first discovered ferroptosis inducer, exhibited limited efficacy in clinical trials, largely due to its inefficient uptake by cancer cells [145, 256]. Combining sigma-2 ligands with demethylated erastin has proven to be a promising strategy as it significantly enhances drug targeting and uptake. This approach takes the sigma-2 receptor, which is typically overexpressed in solid tumor cells, as the drug target [257–263]. Furthermore, the utilization of nanocarriers can also improve the targeting of ferroptosis inducers while often presenting fewer toxic side effects compared to other administration methods [18, 197]. Moreover, it is essential to explore specific genetic markers or biomarkers associated with ferroptosis induction in both preclinical and clinical cancer settings. This research can aid in the identification of potential side effects related to ferroptosis treatments and help develop strategies to manage and enhance treatment safety.

Secondly, a promising strategy is the combination of ferroptosis inducers with other chemotherapeutic drugs, particularly for overcoming chemotherapy resistance, which is frequently encountered in sarcoma treatment, notably resistance to cisplatin. Additionally, the concurrent use of inducers targeting different cell death modalities, such as ferroptosis and apoptosis, holds the potential for enhanced antitumor effects. Consequently, it is crucial to carefully select suitable ferroptosis-inducing drugs, explore their combination therapies to mediate better effects, and lay a solid theoretical foundation for their clinical application.

Lastly, it’s worth noting that FRGs hold promise in predicting sarcoma prognosis and refining clinical staging, providing a direction for the future research of molecular targeted therapy of sarcoma [9, 235]. However, the lack of research on FRGs, the data set from retrospective study and the limited clinical variable data have affected the accuracy and practicability of this technology. Further research and data collection are needed to enhance their precision and clinical relevance.

## Conclusion

In summary, gaining a comprehensive understanding of the mechanisms underlying ferroptosis is crucial for its effective application in treating various diseases. Ferroptosis holds significant promise in sarcoma treatment by effectively targeting sarcoma cells, reducing chemotherapy resistance, and aiding in prognosis prediction. Future research directions include exploring additional drugs and targets for inducing ferroptosis and identifying novel strategies to enhance sarcoma treatment. However, the field is currently hindered by the lack of well-established theoretical foundations and clinical trials, presenting challenges to the clinical use of ferroptosis inducers. Despite these obstacles, the potential in this area is immense, and further exploration is warranted.

## Data Availability

Not applicable.

## Abbreviations

RSL: RAS-selective lethal small molecule
LIP: Labile iron pool
ROS: Reactive oxygen species
PUFAs: Polyunsaturated fatty acids
LOX: Lipoxygenase
POR: Cytochrome P450 oxidoreductase
TF: Transferrin
TFRC: Transferrin receptor
SLC40A1: Solute carrier family 40 member 1
FPN: Ferroportin
FAO: Fatty acid β-oxidation
LPCAT3: Lysophosphatidylcholine acyltransferase-3
ACSL4: Acyl-CoA synthetase long-chain family member 4
PL-PUFAs: Phospholipid-bound polyunsaturated fatty acids
AA: Arachidonic acid
GSH: Glutathione
GPX4: Glutathione peroxidase 4
NADPH: Nicotinamide adenine dinucleotide phosphate
FSP1: Ferroptosis suppressor protein 1
CoQ10: Coenzyme Q10
GCH1: GTP cyclohydrolase 1
BH4: Tetrahydrobiopterin
SLC7A11: Solute carrier family 7 member 11
SLC3A2: Solute carrier family 3 member 2
GCL: Glutamate-cysteine ligase
Nrf2: Nuclear factor erythroid 2-related factor 2
CARS1: Cysteinyl-tRNA synthetase 1
HMG-CoA: 3-Hydroxy-3-methylglutaryl-coenzyme A
AIFM2: Apoptosis-inducing factor mitochondria-associated 2
TCA: Tricarboxylic acid
α-KG: α-Ketoglutarate
ACSF2: Acyl CoA synthetase family member 2
CS: Citrate synthase
VDAC2/3: Mitochondrial resident voltage-dependent anion channel 2/3
DPP4: Dipeptidyl peptidase-4
AML: Acute myeloid leukemia
NSCLC: Non-small-cell lung cancer
PR-DUB: Polycomb-repressive deubiquitinase
H2A: Histone 2A
EMT: Epithelial-to-mesenchymal transition
TME: Tumor microenvironment
LPO: Lipid peroxides
TAMs: Tumor-associated macrophages
DCs: Dendritic cells
DAMPs: Damage-related molecular patterns
FRGs: Ferroptosis-related genes
IDO: Indoleamine 2, 3-dioxygenase
p-STAT3: Phosphorylated signal transducer and activator of transcription 3
FAC: Ferric ammonium citrate
FAS: Ferrous ammonium sulfate
PEITC: Phenethyl isothiocyanate
HMOX1: Heme oxygenase 1
FANCD2: Fanconi anemia complementation group D2
miRNA: MicroRNA
lncRNA: Long non-coding RNA
PDT: Photodynamic therapy
MPPa-PDT: Pyropheophorbide-α methyl ester-mediated PDT
YAP: Yes-associated protein
RMS: Rhabdomyosarcoma
ERK: Extracellular signal-regulated kinase
PKC: Protein Kinase C
NOX: NADPH Oxidase
ES: Ewing sarcoma
AURKA: Aurora kinase A
CA: Carbonic anhydrase
IDH: Isocitrate dehydrogenase
HSF1: Heat shock factor 1
BMDCs: Bone marrow-derived dendritic cells
MDSCs: Myeloid-derived suppressor cells
PLGA: Poly (lactic-co-glycolic) acid
UA: Ursolic acid
STS: Soft tissue sarcoma
ICGs: Immune checkpoint genes
